# Eosinophil Secretion of Granule-Derived Cytokines

**DOI:** 10.3389/fimmu.2014.00496

**Published:** 2014-10-27

**Authors:** Lisa A. Spencer, Kennedy Bonjour, Rossana C. N. Melo, Peter F. Weller

**Affiliations:** ^1^Department of Medicine, Beth Israel Deaconess Medical Center, Harvard Medical School, Boston, MA, USA; ^2^Laboratory of Cellular Biology, Department of Biology, Federal University of Juiz de Fora (UFJF), Juiz de Fora, Brazil

**Keywords:** secretion, eosinophil, granule, degranulation, piecemeal degranulation, cytolysis, cytokine

## Abstract

Eosinophils are tissue-dwelling leukocytes, present in the thymus, and gastrointestinal and genitourinary tracts of healthy individuals at baseline, and recruited, often in large numbers, to allergic inflammatory foci and sites of active tissue repair. The biological significance of eosinophils is vast and varied. In health, eosinophils support uterine and mammary gland development, and maintain bone marrow plasma cells and adipose tissue alternatively activated macrophages, while in response to tissue insult eosinophils function as inflammatory effector cells, and, in the wake of an inflammatory response, promote tissue regeneration, and wound healing. One common mechanism driving many of the diverse eosinophil functions is the regulated and differential secretion of a vast array of eosinophil-derived cytokines. Eosinophils are distinguished from most other leukocytes in that many, if not all, of the over three dozen eosinophil-derived cytokines are pre-synthesized and stored within intracellular granules, poised for very rapid, stimulus-induced secretion. Eosinophils engaged in cytokine secretion *in situ* utilize distinct pathways of cytokine release that include classical exocytosis, whereby granules themselves fuse with the plasma membrane and release their entire contents extracellularly; piecemeal degranulation, whereby granule-derived cytokines are selectively mobilized into vesicles that emerge from granules, traverse the cytoplasm and fuse with the plasma membrane to release discrete packets of cytokines; and eosinophil cytolysis, whereby intact granules are extruded from eosinophils, and deposited within tissues. In this latter scenario, extracellular granules can themselves function as stimulus-responsive secretory-competent organelles within the tissue. Here, we review the distinctive processes of differential secretion of eosinophil granule-derived cytokines.

## Introduction

### Eosinophils are distinguished by their eosin-loving specific granules

**Figure 1:**
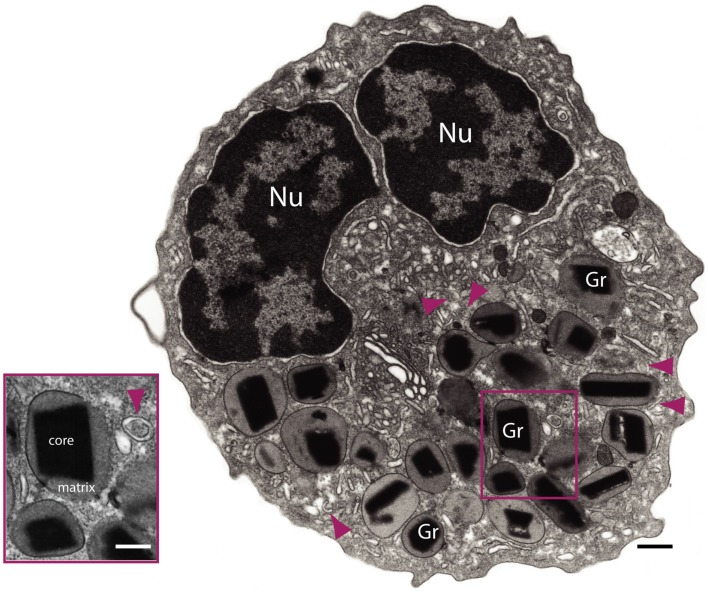
**Transmission electron microscopy of a human eosinophil**. This cell is characterized by a major population of specific granules (Gr) with a unique morphology – an internal often electron-dense crystalline core and an outer electron-lucent matrix surrounded by a delimiting trilaminar membrane. Note the typical bilobed nucleus (Nu) and large tubular carriers (arrowheads). The inset shows secretory granules and a tubular vesicle at higher magnification. Bars: 500 nm; 300 nm (inset).

Paul Ehrlich’s discovery of eosinophils in 1879 was based on the distinctive “eosin-loving” property of eosinophil intracellular granules. The characteristic dark pink punctate staining seen in standard hematoxylin and eosin (H&E) preparations is due to the high cationic protein content of eosinophil granules reacting with the acid dye eosin (1). The most abundant (and most cationic) of the eosinophil granule-derived proteins is major basic protein (MBP), and it is MBP that forms the crystalline lattice structure of the eosinophil granule core, an identifying ultrastructural feature of eosinophils (Figure 1). Eosinophils store their hydrolytic enzymes and cationic granule proteins, including MBP, eosinophil cationic protein (ECP), eosinophil peroxidase (EPO), and eosinophil-derived neurotoxin (EDN), within the core and surrounding matrix of eosinophil specific granules (Figure 1), and it has been long appreciated that secretion of these granule-derived proteins can exert toxic effects on parasites, microbes, and host tissue cells [reviewed in Ref. (2)].

More recently appreciated is that in addition to cationic proteins and hydrolytic enzymes, eosinophils are sources of numerous (over three dozen identified to date) cytokines and chemokines, with a range of biological functions (3, 4). It is now recognized that along with the cationic proteins, many, if not all, of these cytokines are stored within eosinophil specific granules, available for very rapid secretion without the need for *de novo* synthesis (5). A recent study demonstrated co-expression of at least seven immunomodulatory cytokines preformed within specific granules of human blood eosinophils (6), and a number of physiological stimuli have been identified that elicit differential secretion of granule-stored cytokines from eosinophils (7–10). Therefore, it is fitting that the distinguishing morphological feature of eosinophils (i.e., their specific granules) should also represent a functional distinction for these cells.

### Vast array and biological relevance of eosinophil granule-derived mediators and mechanisms of secretion

With the growing awareness of the diverse repertoire of eosinophil granule-derived cytokines has come an evolution in understanding the varied roles eosinophils play in biology. Previously considered strictly end-stage effector cells in parasitic helminth infections and allergic diseases such as asthma, eosinophils, and their secreted products are now regarded as participants in organ development (11, 12), metabolism (13), maintaining (14–16) and/or recruiting (17) lymphocyte populations, anti-microbial (18–22) and fungal (23–25) immunity, tissue repair and regeneration (26–31), immunomodulation (32–37), and tumor immunity (38), and reviewed in Ref. (39).

**Figure 2:**
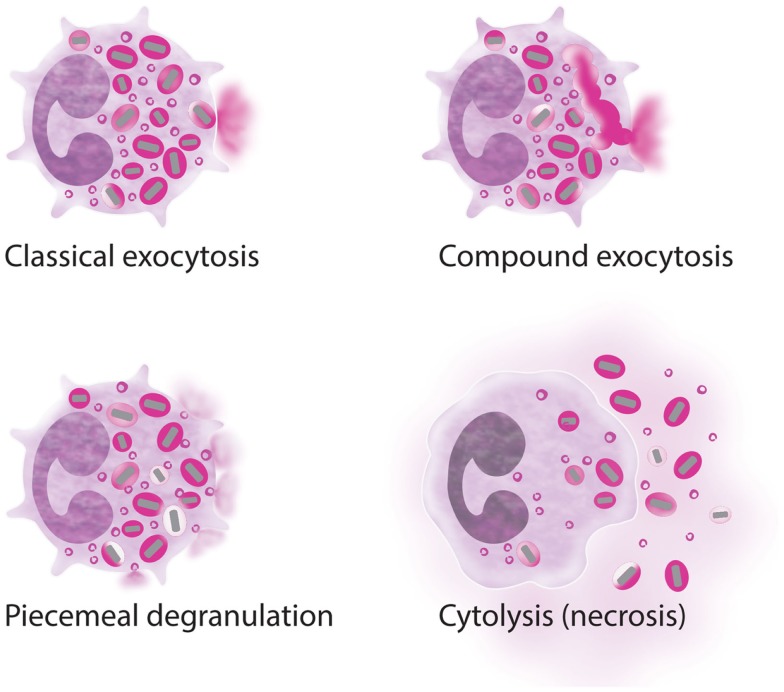
**Processes of eosinophil secretion**. Eosinophils may secrete their granule proteins by classic exocytosis (individual granule fusion with the plasma membrane and release of the total granule content); compound exocytosis (intracellular granule–granule fusion before extracellular release); piecemeal degranulation (vesicular transport of small packets of materials from the secretory granules to the cell surface); and/or cytolysis (extracellular deposition of intact granules upon cell lysis). More than one process can be involved in inflammatory responses.

How does the eosinophil accomplish the highly selective process of secretion of its granule-derived proteins? Basic Immunology textbooks often define degranulation from granulocytes such as eosinophils to occur by a process of classical exocytosis, whereby intracellular granules fuse with the plasma membrane and engage in a wholesale release of granule contents, or in more extreme instances compound exocytosis, whereby intracellular granules fuse together prior to fusion with the plasma membrane and release of their combined contents (Figure 2). Although degranulation via classic and compound exocytosis is observed upon interaction with very large metazoan parasites, in most other physiologically relevant scenarios eosinophils either (1) differentially and progressively secrete their granule-stored contents through a vesicle-dependent process termed piecemeal degranulation (PMD) or (2) deposit intact granules directly into the tissue through a distinctive mode of cell death, termed eosinophil cytolysis (Figure 2). To appreciate the extensiveness of PMD and cytolysis in tissue eosinophils *in situ*, we would refer the reader to Erjefalt et al. (40), and Saffari et al. (41), wherein using morphological criteria the authors quantify the number of tissue eosinophils undergoing PMD and/or cytolysis in association with allergic diseases (i.e., allergic rhinitis and asthma), eosinophilic esophagitis (EoE), or inflammatory bowel diseases (IBDs). In the former study, nearly all eosinophils from tissue sections of allergic disease patients exhibited evidence of PMD, and 27% of the eosinophils showed signs of cytolysis. In IBD samples, greater than 50% of the tissue eosinophils exhibited signs of PMD, while 14% were cytolytic (40). In the latter study, approximately 93% of esophageal eosinophils in EoE exhibited two or more features of degranulation, including loss of cell membrane integrity (marker of cytolysis), cytoplasmic vesiculation (PMD), or reversal of granule core staining (PMD). A total of 70% of esophageal eosinophils exhibited all three features (41). In the remainder of this manuscript, we will explore these two distinct modes of eosinophil secretion of granule-derived proteins.

## Piecemeal Degranulation

The process of PMD was first identified ultrastructurally through electron microscopy studies of mast cells, basophils, and eosinophils in the mid 1970s (42). PMD is characterized by a progressive emptying of granule contents without granule to plasma membrane fusions, rather PMD is accomplished by numerous spherical and tubular secretory vesicles that shuttle granule-derived proteins from the granule to the plasma membrane for secretion. Within the past decade, biochemical and advanced microscopic techniques have enabled an unprecedented look into the process of PMD, and are revealing eosinophil intracellular granules to be dynamic organelles, which undergo protein sorting and vesicle formation.

### Eosinophil granules are dynamic intracellular organelles

**Figure 3:**
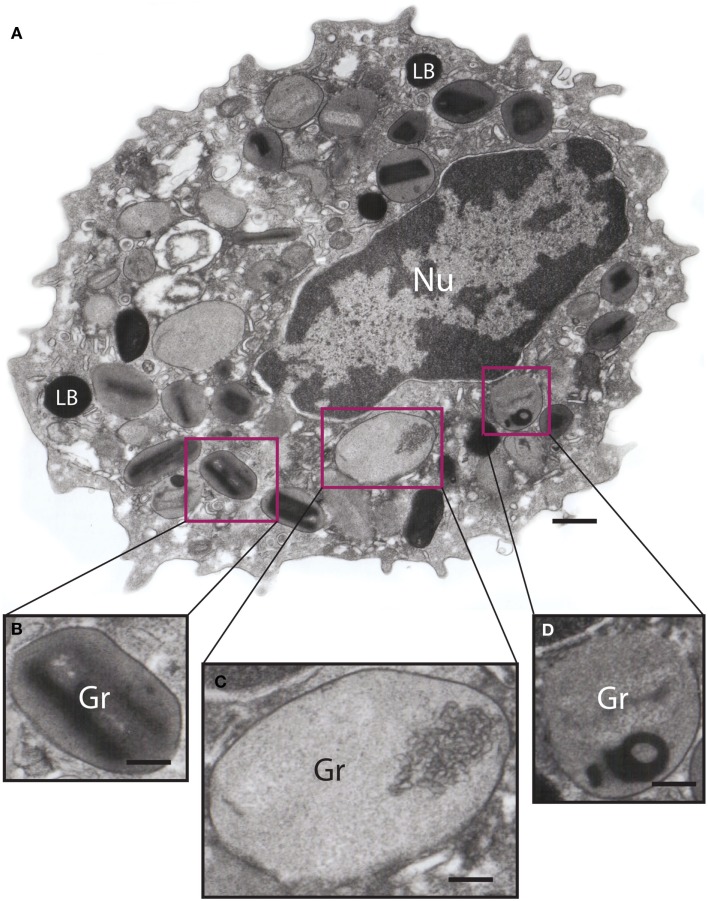
**Ultrastructure of an eotaxin-activated human eosinophil showing piecemeal degranulation (PMD)**. **(A)** After stimulation, specific granules (Gr) exhibit different degrees of emptying of their contents and morphological diversity indicative of PMD, such as **(B)** lucent areas in their cores, **(C)** enlargement and reduced electron density, and **(D)** residual cores. Eosinophils were isolated by negative selection from healthy donors, stimulated with eotaxin-1 for 1 h, immediately fixed and prepared for transmission electron microscopy as before (43). Nu, nucleus; LB, lipid body. Scale bar: 500 nm **(A)**; 170 nm **(B–D)**.

**Figure 4:**
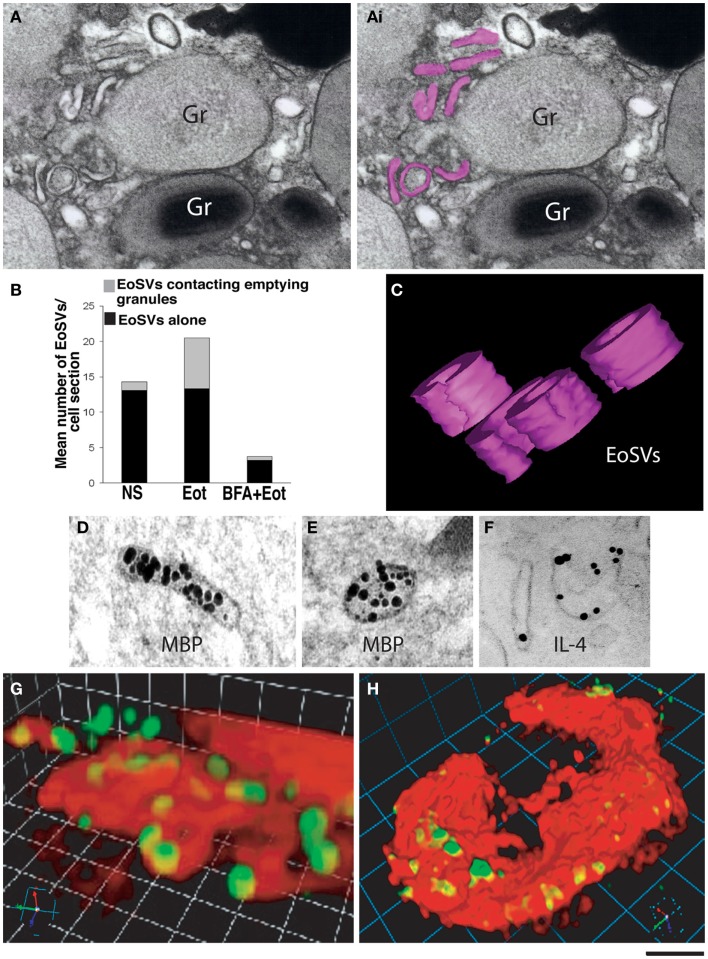
**Vesicular trafficking of granule-derived products from human eosinophils**. **(A)** Eosinophil sombrero vesicles – EoSVs – [highlighted in pink in **(Ai)**] are observed in the cytoplasm surrounding an emptying, enlarged secretory granule (Gr). An intact granule (Gr) with typical morphology is also observed. **(B)** Quantification of EoSV numbers revealed significant formation of these vesicles and association with granules undergoing release of their products, after eotaxin-1 (EOT) stimulation (45). Brefeldin-A (BFA) pretreatment suppressed all EoSV numbers dramatically (*P* < 0.05). NS, not stimulated. **(C**) Three-dimensional (3D) models obtained from electron tomographic analyses show EoSVs as curved tubular and open structures surrounding a cytoplasmic center. **(D–F)** As demonstrated by immunonanogold electron microscopy, major basic protein (MBP) **(D,E)** is transported within the EoSVs lumen, while IL-4 mobilization is associated with vesicle membrane **(F)**. In **(G,H)**, human blood eosinophils suspended in an anti-IL-4 capture antibody-containing agarose matrix were stimulated with eotaxin-1. 3D reconstructed images demonstrate released and captured IL-4 as focal fluorescent green spots at the outer surface of the cell membrane (stained in red). **(B,F–H)** were reprinted from Ref. (45) and **(C–E)** from Ref. (46) with permission. Scale bar: 250 nm **(A)**; 150 nm **(C–F)**; 4 μm **(G)**; 6 μm **(H)**.

Among the earliest ultrastructural indications of PMD are alterations within granules; the crystalline core may become less sharply defined, and variations in the electron density of the core and surrounding matrix occur, representative of disassembled matrices and cores and a reorganization of granule contents (Figure 3). Small spherical vesicles and elongated tubule carriers [eosinophil sombrero vesicles (EoSVs), discussed in the next section] are seen budding from emptying granules, and numbers of spherical vesicles and tubular carriers increase within the cell cytoplasm [(43) and Figures 4A–C]. Immuno-electron microscopy using antibody Fab fragments conjugated to very small nano-gold particles confirm budding vesicles contain granule-derived cytokines and cationic proteins [(43–45) and Figures 4D–F]. Vesicles fuse with the plasma membrane and secrete cytokines extracellularly in discreet packets (Figures 4G,H).

Several lines of evidence support the conclusion that these vesicles are actually generated by and emerge from the granules themselves. Vesicular structures are apparent within emptying granules in the early stages of PMD, and treatment with the vesicular transport inhibitor brefeldin-A (BFA) inhibits the number of cytoplasmic vesicles (Figure 4B) and cytokine secretion by PMD, and causes depositions of membrano-lipid deposits within granules (43). Eosinophils undergoing PMD were further studied by dual-axis automated electron tomography, allowing for tracking of granule structures and content in three dimensions. Tomographic reconstructions demonstrate that granule contents within emptying granules are rearranged into intragranular vesiculotubular compartments and mobilized to the granule membrane. Moreover, both elongated tubular and small spherical vesicles containing granule contents were observed emerging directly from the eosinophil granules in tomographic reconstructions (43, 44, 46).

### Tubular carriers and receptor-mediated trafficking of granule-derived cytokines

As noted above, eosinophils contain over three dozen preformed cytokines; most, if not all, of these cytokines are stored within intracellular granules. Eosinophil granule-derived cytokines are differentially secreted in response to exogenous stimulation, indicating that mechanisms must exist to sort granule-stored cytokines into granule-derived secretory vesicles. One mechanism has been described for the specific mobilization of IL-4. Analysis of eosinophil lysates after subcellular fractionation revealed IL-4 receptor alpha chains (IL-4Rα) are enriched within granule- and vesicle-containing fractions. Upon stimulation of eosinophils with eotaxin-1, a chemokine known to elicit PMD of eosinophil granule-stored IL-4, complementary approaches based in immuno-electron microscopy and flow cytometry demonstrated granule-expressed IL-4Rα is mobilized into secretory vesicles in parallel with IL-4 (47). Importantly, antibodies that compete with IL-4 for binding to IL-4Rα failed to detect vesicle-mobilized IL-4Rα, suggesting that the receptor engages IL-4 during loading into secretory vesicles, and remains engaged while traversing the cytoplasm. This conclusion is supported by immuno-EM studies wherein IL-4 detected within secretory vesicles appears to be membrane-bound (47), in contrast to the free luminal expression pattern exhibited by vesicle-contained MBP (48) (Figures 4D–F). Vesicle-carried TGF-α also exhibits a membrane-associated expression pattern (49), and eosinophils express receptors for most (if not all) of the cytokines that they also store, suggesting that receptor-mediated chaperoning of cognate cytokines might be a universal method of regulating eosinophil-derived cytokine secretion.

This observation of receptor-mediated transport of granule-derived cytokines also provides a function for the large tubular carriers (EoSVs) characteristic of eosinophils undergoing PMD. EoSVs represent a distinct vesicle population, distinguishable from smaller spherical vesicles by morphology and subcellular density. EoSVs observed within the cytoplasm by electron microscopy are viewed as elongated tubes, or when curled, may appear to take on the shape of a “c” or a donut ring (Figures 4A,C). A key aspect of EoSV morphology is the large surface area:volume ratio, a conformation that is optimal for a receptor-mediated transport mechanism (44).

Although the number of cytoplasmic vesicles increases in eosinophils undergoing PMD, spherical vesicles and EoSVs are also observed in non-stimulated eosinophils (see Figure 1). For example, a substantial pool of MBP-loaded vesicles can be observed in intimate association with secretory granules in unstimulated eosinophils (48). It is unclear whether cargo-laden cytoplasmic vesicles are vestiges of a previous round of PMD, and/or whether eosinophils might utilize secretory vesicles as another, rapidly mobilizable, depot for intracellular cytokine storage. Of note, this issue might be relevant to eosinophil postmortem function as well, as we will later see that EoSVs are released along with cell-free granules from cytolytic eosinophils (see Cytolysis below).

### Intracrine regulation of eosinophil PMD

Despite large strides in delineating dynamic intragranule vesiculation and receptor-mediated cytokine sorting, how exogenous signals are transmitted to and decoded by intracellular granules remain unclear. Exposure of eosinophils to a number of physiological stimuli, such as chemotactic lipids and chemokines, cytokines (e.g., IL-3, IL-5, and GM-CSF), and complement components can result in their priming, effectively lowering the signaling threshold for inducing subsequent stimulus-induced cytokine secretion. Intracellular mechanisms that drive eosinophil priming upstream of enhanced secretion are not fully understood. However, inside-out signaling that upregulates the expression, affinity, and/or valency of eosinophil-expressed integrins (e.g., α_M_β_2_) appears to play a significant role [reviewed in Ref. (50)] and may be mediated through a pathway involving PKCβII-dependent phosphorylation of the actin bundling protein l-plastin (51).

In addition to a role for priming in eosinophil secretion, data suggest the existence of intracrine mediators that act on intracellular receptors, possibly expressed on granules. For example, eotaxin-1-induced secretion of IL-4 is dependent upon an intracrine pathway involving the lipid mediator leukotriene C_4_ (LTC_4_). Eotaxin-1 stimulation of eosinophils elicits the generation of LTC_4_ from intracellular lipid bodies; this LTC_4_, acting via an intracellular receptor, is necessary for subsequent IL-4 release (52, 53). Eosinophil intracellular granules express leukotriene receptors on their outer granule membranes (54). Further studies are necessary to determine the specific intracellular target(s) of the LB-generated LTC_4_.

In addition to the intracrine signaling mediators, cytoskeletal elements, GTPases, and membrane-associated proteins further co-ordinate granule and vesicle trafficking, and membrane fusions in PMD. For example, specific SNARE proteins [Soluble NSF Attachment Protein (SNAP) receptors] expressed by granule, vesicle, and plasma membranes within eosinophils co-ordinate membrane tethering, docking, and fusions [reviewed in Ref. (55)]. SNARE-mediated membrane interactions are discussed in more detail in another article within this thematic issue.

## Cytolysis

Intriguingly, secretion of eosinophil granule-derived mediators does not necessarily cease upon cell death. The realization that eosinophils can undergo a distinct mode of cell death that results in the expulsion of intact intracellular granules has been a long time in coming. Structures resembling eosinophil cell-free granules appeared in drawings and stainings of asthmatic sputum as early as the 19th century, and in the early 20th century, free eosinophil granules were observed within pulmonary tissues from fatal asthma, a portion of which were attributed to eosinophil death [reviewed in Ref. (56)]. Importantly, in the late 1990s, Persson et al. helped to validate these earlier descriptions by demonstrating the existence of eosinophil cell-free granules in guinea pig trachea after provocation (i.e., epithelial shedding), using a methodology that could not be discounted on the basis of mechanical artifact, that of performing deep tissue staining of whole mounts (57). However, it is only within the last decade that cytolysis has been more widely appreciated as a physiologically significant mode of eosinophil activity, defined ultrastructurally, and evaluated within the context of specific diseases.

### Eosinophil cytolytic cell death deposits granules, both free and associated with nuclear DNA nets, into surrounding tissue

**Figure 5:**
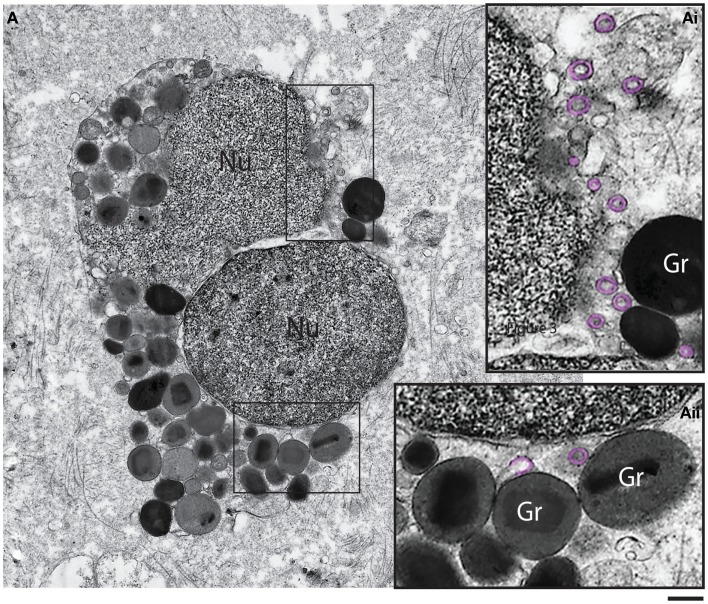
**Ultrastructure of a tissue human eosinophil undergoing cytolysis**. Note the disintegrating nucleus (Nu) and extracellular free secretory granules (Gr) in the surrounding tissue. **(Ai, Aii)** are boxed areas of **(A)** seen at higher magnification. Note the presence of free, intact eosinophil sombrero vesicles (EoSVs – highlighted in pink) in the tissue, after cell lysis. Tissue eosinophils were present in a biopsy performed on a patient with inflammatory bowel disease. Scale bar: 800 nm **(A)**; 300 nm **(Ai, Aii)**.

In addition to eosinophils exhibiting morphological evidence of PMD, micrographs of diseased tissues reveal eosinophils undergoing a cytolytic process of cell death morphologically distinct from both apoptosis and necrosis (58, 59). In contrast to the chromatin condensation and fragmentation of apoptotic nuclei, cytolytic eosinophils are characterized by dissolution of the nuclear membrane and DNA de-condensing into the surrounding cytoplasm (Figure 5). Membrane blebbing characteristic of necrotic cells also does not occur, rather cytolytic eosinophils are typified by a loss of membrane integrity, and release of intracellular contents, including eosinophil specific granules, into the surrounding tissue. Of note, EoSVs are also expelled from cytolytic eosinophils and deposited within the tissue alongside cell-free granules [Figure 5 and (41)]. Tissue-deposited, eosinophil cell-free granules are observed both within the spatial limits of the original cell and also scattered, independently or in clusters, outside of the confines of the originating cell.

The morphological sequelae associated with eosinophil cytolysis are elicited *in vitro* by a number of stimuli, including exposure to a Ca^2+^ ionophore, immobilized IgG or IgA, PMA, and GM-CSF or IL-5 in combination with PAF (58, 60, 61). Closer examination of eosinophil death induced by cross-linking Siglec 8 (62) or exposure to *Staphylococcus aureus* supernatant (63) might implicate these as eosinophil cytolytic stimuli as well (64). By analyzing eosinophils undergoing cytolytic cell death *in vitro* induced by the calcium ionophore A23187, Ueki et al. recently reported a sequence of events that included, chronologically (1) alterations in nuclear shape and density, (2) expulsion of single or small clusters of granules from the cell, (3) decondensation of nuclear contents into the cytoplasm, and (4) loss of membrane integrity, accompanied by the release of single granules or granule clusters (58).

Ueki et al. also demonstrated that under these conditions, eosinophil cytolysis was accompanied by extrusions of nuclear DNA nets. Cell-free granules liberated from cytolytic eosinophils were observed both incorporated into the DNA net-like lattices, and also standing alone as DNA-free granule clusters (58). One might speculate that this so-called “DNA trap cell death” serves a protective function by bringing the anti-microbial power of eosinophil granule-derived proteins into close proximity to pathogens immobilized by a DNA trap. Of note, eosinophil cytolytic DNA trap cell death is reminiscent of the anti-microbial DNA traps described by Yousefi et al. (22) and Morshed et al. (65), wherein mitochondrial DNA is catapulted from live eosinophils along with granule-derived proteins (i.e., ECP and MBP), forming extracellular nets with demonstrated microbicidal functions. However, two important distinctions exist between the DNA nets elicited through eosinophil cytolytic death and the DNA traps described by Yousefi and Morshed. First, in contrast to eosinophil cytolysis-generated nets, the DNA traps described by Yousefi and Morshed emerge from eosinophils that remain viable. Second, the origin of the catapulted DNA in the latter case is mitochondrial, while cytolytic eosinophils extrude DNA nets of nuclear origin. Stimuli causing the expulsion of mitochondrial DNA traps from viable eosinophils include brief stimulation of IL-5-, or IFN-γ-primed eosinophils with LPS, C5a, or eotaxin (22), or stimulation of non-primed eosinophils with TSLP (65). It has yet to be seen how, if at all, the processes of DNA net extrusion from cytolytic eosinophils and the expulsion of mitochondrial DNA nets from viable eosinophils might relate to one another.

### Some eosinophil granules extruded from cytolytic eosinophils remain secretory-competent organelles

One might predict that the consequence of extracellular granule release through eosinophil cytolysis, whether in association with or distinct from DNA nets, would be the continued capacity of eosinophils to deliver their granule contents postmortem. Micrographs of diseased tissues reveal eosinophil extracellular granules exhibiting varying degrees of dissolution of their delimiting membranes (56), suggesting some fraction of cell-free granules with compromised granule membrane integrity might “leak” their protein content within tissues. However, very recent studies now indicate that a portion of extracellularly deposited granules retain the integrity of an intact granule membrane (58). Moreover, extracellularly deposited granules express chemokine, cytokine, and lipid receptors on their outer membrane, such that the ligand binding domains are outwardly oriented and thereby available to interact with exogenous stimuli within the tissue [reviewed in Ref. (66)]. Neves et al. demonstrated outwardly oriented granule-expressed receptors to be functional; in response to exogenous eotaxin-1, IFN-γ, or leukotrienes, eosinophil cell-free granules exhibited kinase phosphorylation suggestive of activation of signal transduction pathways within the granule, and differentially released cationic proteins and cytokines in a stimulus dose- and kinase-dependent manner (54, 67). The implication of these findings is that cell-free granules liberated from cytolytic eosinophils function as stimulus-dependent, secretory-competent organelles within tissues.

## Concluding Remarks and Future Directions

Biological functions of eosinophil-derived cytokines are a burgeoning field. Earlier views of eosinophils as strictly end-stage effectors in parasitic diseases are now being expanded to encompass a new understanding of eosinophils as multifunctional leukocytes participating in developmental, metabolic, and immune cell functions. Keeping pace with newly appreciated eosinophil functions in heath and disease is a growing understanding of the dynamic complexities involved in the stimulus-dependent, differential liberation of cytokines from eosinophil intracellular granules, both through the vesicular transport-based process of PMD in viable eosinophils, and postmortem through tissue-deposited eosinophil cell-free granules and EoSVs elicited from cytolytic eosinophils. These cutting edge mechanistic insights will be critical to the next generation of therapeutic approaches in targeting eosinophil-associated diseases, where one must now consider the manner by which eosinophils die when devising anti-eosinophil strategies, and must measure contributions of tissue-deposited eosinophil cell-free organelles when evaluating cytokine-mediated functions of eosinophils *in situ* in disease.

## Conflict of Interest Statement

The authors declare that the research was conducted in the absence of any commercial or financial relationships that could be construed as a potential conflict of interest.
